# Paleoserology points to Coronavirus as possible causative pathogens of the ‘Russian flu’

**DOI:** 10.1111/1751-7915.14058

**Published:** 2022-04-05

**Authors:** Lindsay Ramassy, Hamadou Oumarou Hama, Caroline Costedoat, Michel Signoli, Emeline Verna, Bernard La Scola, Gérard Aboudharam, Rémi Barbieri, Michel Drancourt

**Affiliations:** ^1^ IHU Méditerranée Infection Marseille France; ^2^ IRD, MEPHI, IHU Méditerranée Infection Aix‐Marseille University Marseille 13005 France; ^3^ CNRS, EFS, ADES Aix‐Marseille University Marseille France

We have read with great interest the paper related to the aetiology of the ‘Russian flu’, that you recently edited and published in Microbial Biotechnology (Brüssow and Brüssow, 2021). In this paper, the authors reviewed epidemiological and clinical data published by the English and German contemporaries of the ‘Russian flu’, a deadly pandemic that occurred in continental Europe between 1889 and 1891 (Valleron *et al*., 2010). ‘Russian flu’ appeared in Bukhara, Uzbekistan in May 1889 (Sisley, 1891) and spread around the world via steamboat and railroad in at least three waves between 1891 and 1893 (Brüssow and Brüssow, 2021) killing an estimated total of one million people in Europe only (Honigsbaum, 2013).

This new reading of the historical medical documents published in Great Britain and Germany, raised the hypothesis of a Coronavirus pandemic at the end of the 19th century: retrospective analyses showed that historical descriptions of ‘Russian flu’ were characterized by intestinal, respiratory and neurological signs specifically including loss of taste and smell, similar to what was described during the current COVID‐19 pandemic caused by a SARS‐CoV‐2 Coronavirus (Brüssow and Brüssow, 2021). However, the aetiology of the ‘Russian flu’ remains controversial in the absence of any direct or indirect paleomicrobiological diagnosis, and the hypothesis of an influenza *stricto sensu* caused by an Influenza virus has also been proposed (Dowdle, 1999).

We have recently applied paleo serological methods that we have previously developed (Oumarou Hama *et al*., 2020) to the exploration of male individuals who died from war‐related injuries in August 1914 in Spincourt (Meuse, France) at the very beginning of the First World War; and who have potentially been exposed to ‘Russian flu’ on the basis of their birth date in France, between 1864 and 1894 (Verna *et al*., 2020) (Table 1). The paleoserological methods we used, were based on extraction and characterization of immunoglobulins from the dental pulp contained in the teeth of deceased and buried individuals. Indeed, dental pulp contained dried blood as it was at the time of the individual's death (Barbieri *et al*., 2017, 2020). Specifically, in this work, the mini‐line blot method was applied to 29 pulp samples collected from 29 deceased individuals in Spincourt, as previously described (Raoult and Dasch, 1989; Oumarou Hama *et al*., 2020). In the presence of a negative control consisting of skimmed milk, we tested the presence of antibodies against Coronaviruses including HCoV‐229E alpha‐Coronavirus, HCoV‐OC43 beta‐Coronavirus and SARS‐CoV‐2 beta‐Coronavirus produced on cell culture tested negative for *Mycoplasma* spp. Coronavirus were heat‐inactivated, a procedure shown to preserve antigenicity of the major Coronavirus antigens, specifically the spike protein antigenicity, as previously described (Edouard *et al*., 2021). While negative controls remained negative, 1/29 paleoserum sample collected from soldier 521 showed reactivity against the Influenza viruses A and B contained in the 2020 vaccine (FluarixTetra, GSK vaccines, Brentford, UK) and 5/29 (24%) collected from soldiers 500, 508, 511, 512, 528 showed anti‐Coronavirus reactivity; exhibiting area under curve > 500 units (determined on the basis of the negative and positive control area under curve values) against SARS‐CoV‐2 in one sample, 229E in two samples and OC43 in four samples; with the individual 528 exhibiting a significant reactivity against the three Coronaviruses (Fig. 1).

**Table 1:** Ages of seven individuals investigated in this study.

Individual	Age class (years)^a^
500	30–59
508	30–59
511	20–39
512	30–59
521	20–49
528	20–49
533	30–59

^a^ Age was estimated via the evaluation of pubic symphysis morphology (Schmitt, 2008) and auricular surface of the pelvic bone (Schmitt, 2005), in agreement with the French Army archives indicating that these soldiers were between 20 and 50 years (Verna *et al*., 2021).

**Fig. 1:**
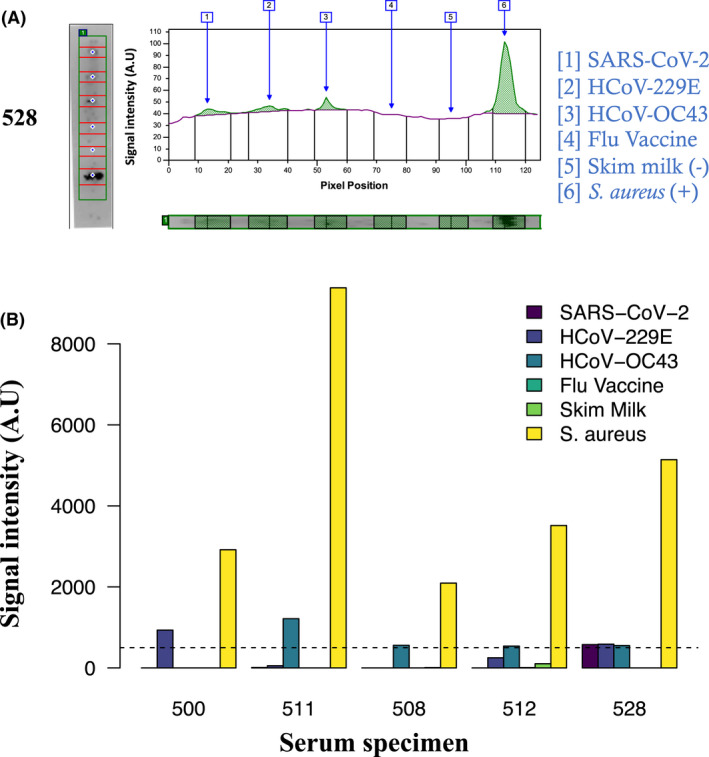
Coronavirus and Influenza virus paleoserology assay in soldiers died in Spincourt, France, 1914. Panel A shows an exemplary mini‐line blot assay performed for individual 508 (left panel) and corresponding quantification obtained by the FUSION FX chemiluminescent imaging system and the ImageQuant TL software (Witec AG, Sursee, Switzerland) (right panel). The *x*‐axis indicated the coordinates of the pixel position, as surrogates for the antigen; the *y*‐axis indicated the intensity of the serological signal in arbitrary units. Panel B presents area under the curve (A.U) values > 500 (represented by a dotted line) obtained after mini‐line blot assay for each antigen tested, in five individuals.

These very preliminary experimental results of a few samples support the hypothesis that a Coronavirus was responsible for the ‘Russian flu’, as derived from a review of the medical historical texts recently published (Brüssow and Brüssow, 2021). Incorporation of pre‐1890 samples may not guarantee them as negative controls, in the absence of further data regarding the antiquity of Coronaviruses and the absence of Coronavirus circulating in populations before 1890. However, due to extensive cross‐reactivity between Coronaviruses, mainly supported by the relatively conserved nucleocapsid protein antigenicity, it was not possible to derive from our data, the exact Coronavirus species implied in the ‘Russian flu’. Therefore, it is necessary to consolidate these indirect diagnostic data by incorporation of recombinant Coronavirus antigens into the mini‐line blot assay; and in case of suspected ancient viremia to attempt direct diagnosis based on the detection of specific Coronavirus peptide sequences using paleoproteomics methods (Barbieri *et al*., 2017), or even nucleotide sequences by metagenomics, in order to support the Coronavirus hypothesis in the controversial aetiology of ‘Russian flu’.

## Conflict of interest

The authors have no conflicts of interest to declare.
